# A multi-stage validation of the C-reactive protein–triglyceride–glucose index for predicting mild cognitive impairment: evidence from clinical and nationwide prospective cohorts

**DOI:** 10.3389/fendo.2026.1841431

**Published:** 2026-05-19

**Authors:** Yiwen Chen, Jie Sun, Xinyue Chen, Sijue Yang, Xiangguo Cong, Zhijuan Ge, Zhou Zhang, Yan Bi

**Affiliations:** 1 Department of Endocrinology, Endocrine and Metabolic Disease Medical Center, Nanjing Drum Tower Hospital Clinical College of Nanjing University of Chinese Medicine, Nanjing, China; 2 Branch of National Clinical Research Centre for Metabolic Diseases, Nanjing, China; 3 Department of Endocrinology, Endocrine and Metabolic Disease Medical Center, Nanjing Drum Tower Hospital, Affiliated Hospital of Medical School, Nanjing University, Nanjing, China; 4 Department of Endocrinology, Endocrine and Metabolic Disease Medical Center, Nanjing Drum Tower Hospital Clinical College of Nanjing Medical University, Nanjing, China

**Keywords:** C-reactive protein-triglyceride-glucose index, cross-sectional study, mild cognitive impairment, prediction model, type 2 diabetes mellitus

## Abstract

**Background:**

Mild cognitive impairment (MCI) is a common but under-recognized complication of type 2 diabetes mellitus (T2DM), and practical predictors are limited. The C-reactive protein–triglyceride–glucose index (CTI) integrates metabolic and inflammatory status and may better capture the pathways underlying cognitive decline.

**Methods:**

The study included 1, 580 patients with T2DM as the primary cohort and utilized the China Health and Retirement Longitudinal Study (CHARLS, n = 4, 575) for external validation. Variables were selected using LASSO and stepwise regression. The area under the curve (AUC), net reclassification improvement (NRI), and integrated discriminant improvement (IDI) were used to evaluate the model’s performance, while restricted cubic splines (RCS) were used to investigate the dose-response connection.

**Results:**

In both the main cohort (OR = 1.16, 95% CI: 1.04–1.30) and the CHARLS cohort (HR: 1.245, 95% CI: 1.164–1.332), a high CTI score was independently associated with an increased risk of cognitive impairment. In the main cohort, the CTI significantly outperformed the TyG index in terms of risk reclassification (NRI = 0.132, P = 0.031). RSC analysis revealed a significant linear relationship in individuals with normal glucose levels and prediabetes. Furthermore, the ROC curve developed in this study demonstrated good calibration and clinical net benefit.

**Conclusions:**

The CTI index is a robust composite marker for predicting the risk of MCI. It has strong predictive value in the early stages of impaired glucose metabolism, making it a useful tool for identifying high-risk individuals and delivering customized therapies.

## 1 Introduction

With the rapid aging of the global population, age-related cognitive decline has become an increasingly pressing public health issue (1, 2). Among older adults, individuals with type 2 diabetes mellitus (T2DM) face a particularly high risk of developing mild cognitive impairment (MCI) and dementia (3). In this context, identifying those at elevated risk at an early stage is essential, not only for timely intervention but also for improving the allocation of healthcare resources.

A growing body of evidence suggests that cognitive impairment in T2DM arises from the combined effects of insulin resistance and chronic low-grade inflammation, two interrelated and potentially modifiable processes (4–6). The triglyceride-glucose (TyG) index, obtained from fasting glucose and triglyceride levels, has been widely employed as a surrogate diagnostic of insulin resistance and has demonstrated links with cognitive impairment (7). Similarly, lower cognitive results have been associated with C-reactive protein (CRP), a well-known indicator of systemic inflammation (8). However, each of these markers reflects only one side of a more complex biological process. The interaction between metabolic dysfunction and inflammation is likely to play a central role, yet it is not fully captured by any single indicator (9).

Despite increasing recognition of this interplay, most previous studies have examined metabolic or inflammatory markers in isolation. As a result, the potential advantage of combining these pathways into a single, integrated measure remains underexplored. A recent study by Ding et al. (10) proposed a clinical model for predicting MCI based on demographic and clinical characteristics. While such approaches are useful, incorporating objective biochemical markers may further improve risk stratification.

The C-reactive protein–triglyceride–glucose (CTI) index has recently been introduced as a composite measure that integrates both systemic inflammation and insulin resistance (11). By design, it captures the combined metabolic–inflammatory burden, rather than focusing on a single pathway. Emerging evidence has linked CTI to a range of conditions, including cardiovascular disease (12), non-alcoholic fatty liver disease (13), and depressive symptoms (14), suggesting that it may reflect broader systemic dysregulation. Importantly, CTI is derived from routine laboratory tests, making it both accessible and feasible for use in clinical and population-based settings.

Against this background, the present study was designed to examine the relationship between CTI and MCI in a more comprehensive manner. Using a two-stage approach that combines cross-sectional analysis with prospective validation in the CHARLS cohort, we aimed to assess the stability of this association across different glycemic states. In addition, we evaluated whether CTI provides incremental predictive value and improves risk reclassification beyond traditional metabolic markers, with the intention of providing a more useful instrument for the early detection of people who are at risk of cognitive impairment.

## 2 Materials and methods

### 2.1 Research design

This study was designed as a two-stage observational analysis. In the first stage, a cross-sectional dataset was used to examine the association between CTI and MCI. In the second stage, an independent cohort was used to validate the association between CTI and cognitive decline.

### 2.2 Research population

#### 2.2.1 Hospital-based cross-sectional cohort

**Figure 1:**
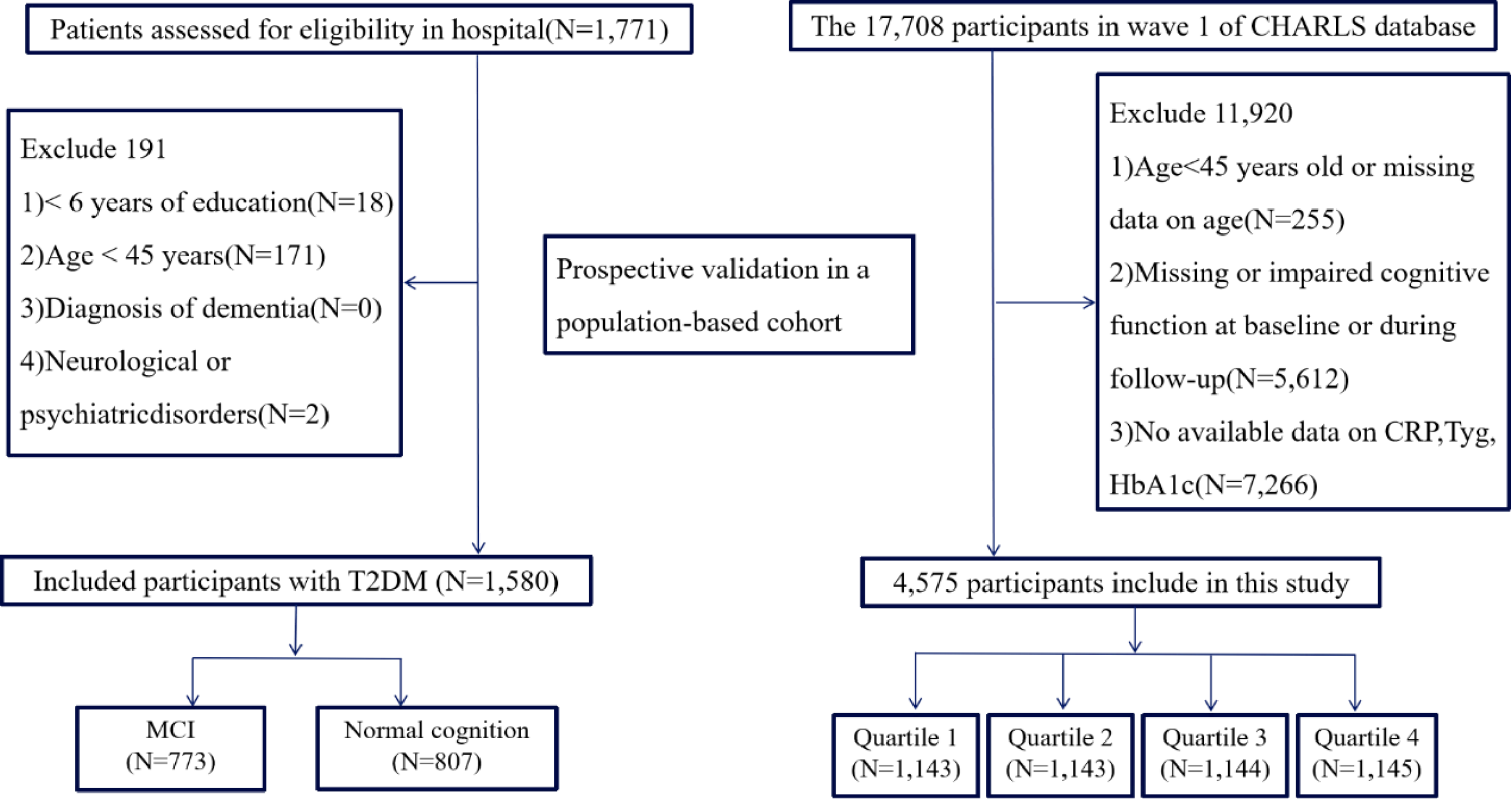
Flowchart of participant selection and study design. Legend: In the hospital-based cross-sectional study, 1, 771 patients were screened, and 1, 580 individuals with type 2 diabetes mellitus (T2DM) were included after applying exclusion criteria. Participants were categorized into mild cognitive impairment (MCI) and normal cognition groups. In the population-based cohort derived from the China Health and Retirement Longitudinal Study (CHARLS), 17, 708 individuals from wave 1 were assessed. After excluding participants with missing data, age <45 years, or impaired or incomplete cognitive assessments at baseline or during follow-up, 4, 575 participants were included in the longitudinal analysis.

A cross-sectional analysis was performed on a series of consecutive T2DM patients who underwent both cognitive assessments and laboratory testing at Nanjing Drum Tower Hospital, Affiliated Hospital of Nanjing University Medical School, between August 2022 and July 2025. To be included in the study, individuals had to meet the following prerequisites: they were at least 45 years old; their T2DM was diagnosed in line with the guidelines from the American Diabetes Association (15, 16); and they were capable and willing to undertake a thorough neuropsychological evaluation. Exclusion criteria included: fewer than six years of formal education; a clinical diagnosis of dementia; and a historical or current diagnosis of neurological or psychiatric disorders. Participants were excluded sequentially according to the predefined criteria, as illustrated in **Figure 1**.

#### 2.2.2 Population-based longitudinal cohort

The data for longitudinal cohort were derived from the China Health and Retirement Longitudinal Study (CHARLS), a nationally representative longitudinal survey of Chinese residents aged 45 and older.

Baseline data were derived from Wave 1 (2011), with follow-up assessments conducted through Wave 4 (2018). From the initial sample of 11708 participants, we excluded individuals with missing CTI data, incomplete cognitive assessment. To establish a temporal relationship and minimize reverse causality, we further restricted our analytical sample to those without evidence of cognitive impairment at study entry. Baseline cognitive impairment was defined as a global cognitive score more than 1 standard deviation below the age and education-adjusted mean, or a self-reported physician diagnosis of memory-related diseases or dementia. After applying these criteria, 4, 575 participants were included in the final analysis. Detailed selection procedures are shown in **Figure 1**.

For the present analysis, participants were further categorized into three glycemic status groups based on baseline measurements. Diabetes was defined as a fasting plasma glucose (FPG) level of 126 mg/dL or higher, HbA1c level of 6.5% or above, or the combination of a self-reported physician diagnosis, usage of hypoglycemic medications, or both. The condition of prediabetes was defined by an FPG of 100 to 125 mg/dL, or an HbA1c of 5.7 to 6.4%. Individuals who did not fit into either the diabetes or prediabetes categories were categorized as having normal glucose regulation (NGR). These definitions are consistent with the American Diabetes Association diagnostic criteria (16).

### 2.3 Cognitive assessment

#### 2.3.1 Cross-sectional cohort

The Mini-Mental State Examination (MMSE) and the Beijing version of the Montreal Cognitive Assessment (MoCA) were used in the cross-sectional investigation to measure global cognitive function. The MMSE was employed for preliminary screening of suspected dementia (17), while the MoCA was used for a more sensitive evaluation of MCI. MCI was diagnosed based on widely accepted criteria: self-reported or informant-confirmed cognitive complaints; objective cognitive impairment as confirmed by clinical evaluation; preservation of basic daily functioning; and absence of a dementia diagnosis (17, 18). For individuals with ≤12 years of education, a 1-point correction was added to the MoCA score. A corrected MoCA score <26 was considered indicative of cognitive impairment (19).

To get a handle on various cognitive abilities, the participants were put through the Repeatable Battery for the Assessment of Neuropsychological Status (RBANS), which takes a close look at five key areas: the ability to remember things right away, skills related to visual space and construction, language capabilities, attention span, and memory recall down the line (20). Meanwhile, processing speed and executive function got their moment in the spotlight using the Trail Making Test (both Parts A and B) along with the Stroop Color and Word Test (Parts I, II, and III). When it came to measuring outcomes, we looked at how many seconds it took folks to complete these tasks, where a longer time clearly pointed to weaker performance. Every single cognitive assessment was carried out by a board-certified neuropsychological examiner in a distraction-free setting, following a carefully laid-out protocol to the letter.

It should be noted that this clinical adjudication process differs fundamentally from the operational definition used in the CHARLS cohort (see Section 2.3.2), where cognitive impairment was defined solely by performance-based criteria. This distinction is important for interpreting the findings across the two cohorts.

#### 2.3.2 Charls cohort

Cognitive function in CHARLS was assessed using a standardized battery covering episodic memory and mental intactness. Episodic memory was evaluated using immediate and delayed word recall tests, and the final score was calculated as the average of the two components. Mental intactness was assessed using items adapted from the Telephone Interview of Cognitive Status (TICS), including orientation, serial subtraction, and figure drawing tasks. A global cognitive score was calculated by summing episodic memory and mental intactness scores.

For the purpose of this study, incident cognitive impairment was operationally defined as a global cognitive score more than 1 standard deviation below the age and education-adjusted mean of the study population at follow-up (18–20). The age- and education-adjustment was performed by regressing the baseline global cognitive score on age and education within each stratum, and the residual-based threshold was applied uniformly at each follow-up wave. Time-to-event was defined as the interval from the baseline interview date to the first follow-up wave at which the participant met the cognitive impairment threshold (21).

It is important to acknowledge that this definition is a practical approximation and does not constitute a clinical diagnosis of Mild Cognitive Impairment (MCI). Unlike the hospital-based cohort, the CHARLS dataset lacks the clinical adjudication required to formally differentiate MCI from other forms of cognitive decline, including dementia. Throughout this manuscript, we use the term “cognitive impairment” when referring to CHARLS-derived outcomes to maintain terminological precision. The implications of this are addressed in sensitivity analyses and the study limitations.

### 2.4 Measurement of CTI Index

Fasting blood samples were collected to measure triglycerides (TG), fasting plasma glucose, and CRP levels. The CTI was calculated as:

CTI = 0.412 × Ln (hs-CRP) + Ln (TG × FPG/2).

This index has been demonstrated to comprehensively reflect the patient’s inflammatory status and metabolic disorder level (22).

### 2.5 Statistical analysis

For each variable, descriptive statistics were calculated. Categorical variables were displayed as counts and percentages, while continuous variables were displayed as mean ± standard deviation (SD) or median (interquartile range, IQR). One-way ANOVA or the Kruskal-Wallis test for continuous variables and the chi-square test for categorical variables were used for between-group comparisons.

Multivariable logistic regression was performed to examine the association between CTI and MCI. To avoid multicollinearity, CTI and its individual components (CRP and TyG index) were analyzed in separate models. Model 1 included TyG, while Model 2 included CTI; both models were adjusted for age, education, diabetes duration, and HbA1c. Adjusted odds ratios (ORs) with 95% confidence intervals (CIs) were reported.

The area under the receiver operating characteristic curve (AUC) was used to evaluate the discriminative performance of CTI for MCI. Net reclassification improvement (NRI) and integrated discrimination improvement (IDI) were used to assess incremental predictive value beyond traditional metabolic indicators. Restricted cubic spline (RCS) regression was applied to explore potential nonlinear associations within the main cohort, using the median CTI as reference.

The relationship between baseline CTI and incident cognitive impairment in the CHARLS cohort was investigated using Cox proportional hazards models for longitudinal validation. CTI was examined using both quartiles and as a continuous variable. Potential confounders such as age, sex, education, BMI, and comorbidities were taken into account by three progressively adjusted models.

To investigate possible effect modification, stratified analyses were performed by age, sex, BMI, and glycemic status. RCS regression was used to further assess threshold effects and nonlinear associations. Schoenfeld residuals were used to confirm the proportional hazards assumption. To test the robustness of our operational definition of MCI and to minimize the potential confounding effect of severe cognitive impairment or dementia, a sensitivity analysis was performed. In this analysis, we further excluded 83 individuals with cognitive impairment accompanied by functional impairment (ADL score > 6) and those with self-reported or clinically diagnosed dementia. The association between the CTI index and MCI was then re-evaluated within this ‘functionally independent’ sub-cohort.

The primary cohort was randomly split into a training set (70%) and an internal validation set (30%). Feature selection was performed using least absolute shrinkage and selection operator (LASSO) regression with 10-fold cross-validation. Variables with non-zero coefficients were subsequently entered into a multivariable logistic regression model.

To evaluate whether alternative modeling approaches could improve predictive performance, we also developed models using random forest and support vector machine (SVM) algorithms. Model performance was assessed by the area under the receiver operating characteristic curve (AUC) in the validation set. Based on a combination of discriminative performance (AUC) and clinical interpretability, the logistic regression model was selected as the primary tool for risk prediction and nomogram construction.

Model calibration was evaluated using calibration plots and the Hosmer-Lemeshow goodness-of-fit test. Clinical utility was assessed using decision curve analysis (DCA). Internal validation was performed using bootstrap resampling with 1, 000 iterations.

To enhance model interpretability, SHAP (Shapley Additive Explanations) analysis was conducted to quantify the contribution and direction of each predictor in the final model. SHAP values provide a unified measure of feature importance and allow for individualized explanation of model predictions.

All statistical analyses were performed using R (version 4.5.2) and SPSS (version 29.0). A two-sided p value < 0.05 was considered statistically significant.

## 3 Results

### 3.1 Baseline characteristics

Among 1, 580 patients with type 2 diabetes mellitus (T2DM), 773 (48.9%) were identified with mild cognitive impairment (MCI). Compared with participants with normal cognition, those with MCI were slightly older (61.1 ± 7.4 vs. 58.3 ± 7.7 years, P < 0.001), more often female (40.3% vs. 29.8%, P = 0.002), and had lower levels of education (P < 0.001). They also exhibited a less favorable metabolic and inflammatory profile, including higher HbA1c (8.76% ± 2.23% vs. 8.32% ± 2.11%, P < 0.001), fasting plasma glucose (8.24 ± 2.83 vs. 7.85 ± 2.51 mmol/L, P = 0.003), and CRP (5.77 ± 14.49 vs. 4.47 ± 8.89 mg/L, P = 0.032). Importantly, the C-reactive Protein-Triglyceride Glucose index (CTI) was higher in the MCI group (7.96 ± 0.80 vs. 7.84 ± 0.76, P = 0.002), and cognitive performance was consistently lower across all domains (all P < 0.001) (**Table 1**). These findings suggest that a higher cumulative metabolic–inflammatory burden may contribute to cognitive impairment.

**Table 1:** Baseline characteristics of participants according to cognitive status in the cross-sectional study.

Variables	MCI(N = 773)	Intact cognition(N = 807)	P value
Demographic			
Age, years	61.12 ± 7.39	58.35 ± 7.72	<0.001
Female, n(%)	311(40.3)	236(29.8)	0.002
Drinking status, n(%)	172(22.7)	227(28.7)	0.007
Smoking status, n(%)	237(31.1)	310(39)	0.001
BMI, kg/m^2^	25.13 ± 4.12	25.48 ± 4.93	0.110
Glycaemic-related index			
Hba1c, %	8.76 ± 2.23	8.32 ± 2.11	<0.001
FPG, mmol/L	8.24 ± 2.83	7.85 ± 2.51	0.003
2-hour PPG, mmol/L	15.38 ± 4.64	15.19 ± 4.34	0.41
Education			<0.001
<9 years	366(47.4)	195(24.2)	
9–12 years	240(31.1)	224(27.8)	
>12 years	166(21.5)	388(48.1)	
Duration			0.001
<5 years	266(34.4)	327(40.5)	
≥5 years	507(65.6)	480(59.5)	
Blood pressure			
Hypertension, n(%)	410(53.7)	377(47.6)	0.017
SBP, mmHg	134.03 ± 19.69	133.84 ± 19.35	0.883
DBP, mmHg	81.36 ± 11.41	82.63 ± 12.86	0.008
Lipid profile			
TC, mmol/l	4.59 ± 1.18	4.66 ± 1.18	0.303
TG, mg/dL	30.03 ± 32.31	28.03 ± 17.41	0.100
HDL, mmol/l	1.212 ± 0.33	1.23 ± 0.34	0.496
LDL, mmol/l	2.72 ± 0.92	2.77 ± 0.95	0.087
Related profile			
CRP, mg/l	5.77 ± 14.49	4.47 ± 8.89	0.032
Tyg	7.45 ± 0.69	7.38 ± 0.66	0.062
CTI	7.96 ± 0.8	7.84 ± 0.76	0.002
Cognitive function			
MOCA score	23.09 ± 2.04	27.07 ± 1.31	<0.001
Visuospatial construction ability	3.67 ± 0.98	4.54 ± 0.661	<0.001
Naming	2.66 ± 0.58	2.93 ± 0.27	<0.001
Attention	5.75 ± 0.57	5.93 ± 0.28	<0.001
Abstract	1.23 ± 0.697	1.75 ± 0.46	<0.001
Delayed memory	1.75 ± 1.352	3.27 ± 1.133	<0.001
Orientation	5.71 ± 0.603	5.92 ± 0.286	<0.001

Values are presented as mean ± standard deviation (SD), median (interquartile range, IQR), or number (percentage), as appropriate. Comparisons between groups were conducted using one-way analysis of variance (ANOVA) or the Kruskal–Wallis test for continuous variables, and chi-square test for categorical variables. BMI, body mass index; HbA1c, glycated hemoglobin; SBP, systolic blood pressure; HDL, high-density lipoprotein; TC, Total Cholesterol; TG, triglyceride; FPG, fasting plasma glucose; CRP, C-reactive protein; LDL, low-density lipoprotein; CTI, C-reactive protein–triglyceride glucose index; TyG, triglyceride glucose index; MoCA, Montreal Cognitive Assessment.

### 3.2 Association between CTI and MCI

#### 3.2.1 Logistic regression analysis

The results of the multivariable logistic regression analysis are presented in **Table 2**. In the fully adjusted model (Model 3), the CTI index was significantly and positively associated with the risk of MCI. Specifically, each 1-standard deviation (SD) increase in the CTI index was associated with a 16% increase in the odds of MCI (OR = 1.16, 95% CI: 1.04–1.30, P = 0.011).

**Table 2:** Association between the CTI index and the prevalence of MCI in the primary cross-sectional cohort.

Categories	Model 1		Model 2		Model 3	
	OR (95% CI)	P value	OR (95% CI)	P value	OR (95% CI)	P value
Continuous variable per unit	1.22 (1.10–1.36)	<0.001	1.18 (1.06–1.32)	0.002	1.16 (1.04–1.30)	0.011
Quartile						
Q1	Ref.		Ref.		Ref.	
Q2	1.26 (0.94–1.68)	0.12	1.31 (0.97–1.77)	0.084	1.29 (0.95–1.75)	0.101
Q3	1.43 (1.07–1.91)	0.016	1.39 (1.03–1.88)	0.034	1.31 (0.96–1.79)	0.085
Q4	1.48 (1.11–1.98)	0.008	1.37 (1.01–1.85)	0.044	1.29 (0.94–1.76)	0.114

Model 1, adjusted for age, gender; Model 2, Added adjustment for Education, Smoke status, Drink status on top of Model 1; Model 3, Added adjustment for hypertension and HbA1c on top of Model 2.

A significant trend was seen in Models 1 (P trend = 0.005) and Model 2 (P trend = 0.041) when CTI was examined as a categorical variable. In Model 1, those in the highest CTI quartile (Q4) were 48% more likely to develop MCI than those in the lowest quartile (Q1) (OR = 1.48, 95% CI: 1.11–1.98, P = 0.008). The point estimates for Q2 (OR = 1.29), Q3 (OR = 1.31), and Q4 (OR = 1.29) consistently stayed above 1.0, indicating a steady positive correlation even if the categorical relationship in Model 3 did not approach statistical significance (P trend = 0.129).

#### 3.2.2 Nonlinear relationship between CTI and MCI

The dose-response relationship between the CTI index and MCI risk was further characterized using restricted cubic splines with four knots. After full adjustment for all covariates in Model 3, the RCS analysis confirmed a significant overall association between CTI and MCI (P overall = 0.01).

**Figure 2:**
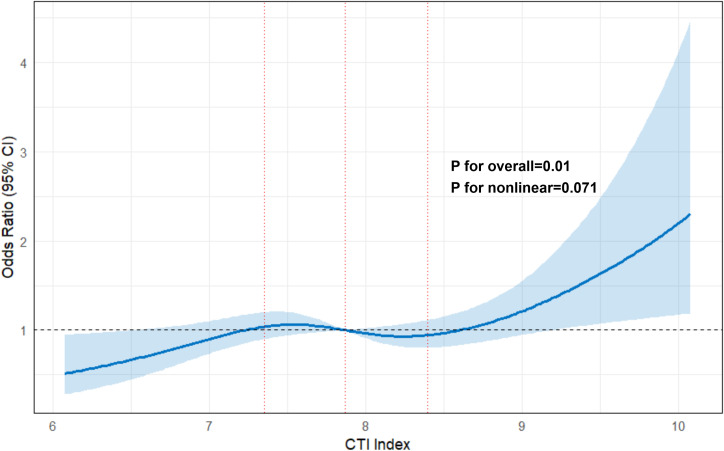
Restricted cubic spline (RCS) curves of the association between the CTI index and the odds of MCI in the primary cohort. Legend: The solid blue line represents the multivariable-adjusted odds ratios (ORs), and the light blue shaded area represents the 95% confidence intervals (CIs). The model was adjusted for age, gender, educational attainment, smoking status, drinking status, hypertension, and HbA1c.

As illustrated in **Figure 2**, the risk of MCI increased progressively with rising CTI levels. The test for non-linearity yielded a P-value of 0.071, indicating that the association between CTI and MCI followed a predominantly linear dose-response pattern rather than a non-linear one. No evidence of a U-shaped or threshold effect was observed, suggesting that the risk increment is stable across the observed range of CTI values in this population.

#### 3.2.3 Subgroup analyses

To evaluate the robustness of the association between the CTI index and MCI risk, stratified analyses were performed across various subgroups based on demographic characteristics, lifestyle factors, and clinical conditions (**Supplementary Figure 1**). The positive association between the CTI index and the odds of MCI remained generally consistent across most analyzed strata. No significant interactions were observed for age (P interaction= 0.361), gender (P interaction = 0.106), smoking status (P interaction = 0.558), drinking status (P interaction = 0.108), BMI (P interaction = 0.559), educational attainment (P interaction = 0.648), diabetes duration (P interaction = 0.992), or hypertension status (P interaction = 0.485).Notably, the CTI index appeared to have a particularly pronounced effect in certain subgroups, such as individuals aged≥60 years (OR = 1.487, 95% CI: 1.215–1.824, P < 0.001) and those with a diabetes duration≥5 years (OR = 1.331, 95% CI: 1.139–1.555, P < 0.001). However, the lack of significant interaction P-values indicates that the CTI index is a stable and independent risk factor for MCI, regardless of the participants’ underlying demographic or clinical profiles.

### 3.3 Incremental predictive value of CTI

To further evaluate whether the CTI index provides superior predictive value over the conventional TyG index for MCI risk, we compared the diagnostic performance of two adjusted models (**Table 3**; **Supplementary Table 1**; **Supplementary Figure 2**). Model 1, which served as the reference, included age, education, diabetes duration, HbA1c, gender and the TyG index. Model 2 replaced the TyG index with the CTI index while adjusting for the same set of covariates. The AUC for Model 2 (0.7066; 95% CI: 0.681–0.732) was numerically higher than that of Model 1 (0.7045; 95% CI: 0.679–0.730), although the difference did not reach statistical significance (P = 0.135). In contrast to the TyG-based model, the model demonstrated a modest but statistically significant improvement in reclassification performance with the addition of the CTI index. Specifically, the category-free Net Reclassification Improvement (NRI) was 0.132 (95% CI: 0.012–0.252, P = 0.031). The Integrated Discrimination Improvement (IDI) was 0.004 (95% CI: 0.001–0.007, P = 0.035), further supporting a limited but statistically significant incremental contribution of the CTI index beyond traditional metabolic and glycemic parameters.

**Table 3:** Comparison of predictive performance between TyG-based and CTI-based models.

Parameters	Model 1 (base + TyG)	Model 2 (base + CTI)	P-value
AUC (95% CI)	0.7045 (0.679–0.730)	0.7066 (0.681–0.732)	0.135
NRI (95% CI)	Reference	0.132 (0.012–0.252)	0.031
IDI (95% CI)	Reference	0.004 (0.001–0.007)	0.035

Model 1, Adjusted for age, education, diabetes duration, HbA1c, and TyG index. Model 2, Adjusted for age, education, diabetes duration, HbA1c, and CTI index. P-value: Comparisons of AUC, NRI, and IDI between Model 2 and Model 1. AUC, area under the curve; NRI, net reclassification improvement; IDI, integrated discrimination improvement; CI, confidence interval.

### 3.4 Prospective validation in the CHARLS cohort

#### 3.4.1 Cox regression analysis

In the unadjusted model (Model 1), the CTI index was significantly associated with a higher risk of cognitive impairment (HR = 1.176, 95% CI: 1.114–1.242, P < 0.001). This association remained highly robust after progressively adjusting for demographic and lifestyle factors in Model 2(HR = 1.219, 95% CI: 1.146–1.298, P < 0.001).

Notably, even after further adjustment for baseline metabolic conditions including hypertension, hyperlipidemia, TC, FPG, HbA1c, and heart disease (Model 3), the CTI index remained a potent independent risk factor for cognitive impairment development. Specifically, each unit increase in the CTI index was associated with a 24.5% increased risk of future cognitive impairment (HR = 1.245, 95% CI: 1.164–1.332, P < 0.001).

A distinct and significant dose-response relationship was found when subjects were divided into quartiles according to their CTI levels. Participants in the higher quartiles showed a steadily rising risk of cognitive impairment when compared to the lowest quartile (Q1, reference).

In the fully adjusted Model 3, individuals in the third quartile (Q3) and the highest quartile (Q4) had a 25.0% (HR = 1.250, 95% CI: 1.064–1.469, P = 0.007) and 63.4% (HR = 1.634, 95% CI: 1.395–1.915, P < 0.001) higher risk of cognitive impairment, respectively. The trend across quartiles was highly significant, reinforcing the evidence that elevated CTI levels at baseline are a strong long-term predictor for the onset of cognitive impairment in the general population. (**Table 4**).

**Table 4:** Hazard ratios (HRs) for incident cognitive impairment according to quartiles of the C-reactive protein-triglyceride glucose index (CTI) in the CHARLS cohort.

Categories	Event, n (%)	Model 1^a^		Model 2^b^		Model 3^c^	
		HR (95% CI)	P value	HR (95% CI)	P value	HR (95% CI)	P value
Continuous variable per unit	1772 (38.7)	1.176 (1.114-1.242)	<0.001	1.219 (1.146-1.298)	<0.001	1.245 (1.164-1.332)	<0.001
Quartile							
Q1	476 (40.86)	Ref.		Ref.		Ref.	
Q2	434 (37.51)	1.155 (1.002-1.332)	0.047	1.166 (0.9935-1.370)	0.061	1.141 (0.97-1.342)	0.112
Q3	402 (35.63)	1.248 (1.085-1.435)	0.002	1.262 (1.076-1.481)	0.004	1.250 (1.064-1.469)	0.007
Q4	416 (36.98)	1.484 (1.297-1.699)	<0.001	1.6 (1.372-1.866)	<0.001	1.634 (1.395-1.915)	<0.001

CTI quartiles: Q1 (<8.211), Q2 (8.211-8.759), Q3 (8.759-9.475), Q4 (>9.475). Model 1, No variables adjusted; Model 2, adjusted for age, BMI, SBP, DBP, gender, educational attainment, smoking habits, and drinking habits; Model 3, Added adjustment for hypertension, hyperlipidemia, TC, FPG, HbA1c and heart disease on top of Model 2. HR, Hazard ratio.

#### 3.4.2 Stratified and nonlinear analysis

To investigate whether the predictive value of the CTI index varied across different glucose metabolic states, we performed a stratified analysis in the CHARLS cohort (**Table 5**). Participants were categorized into three groups: Normal Glucose Regulation (NGR), Pre-diabetes (Pre-DM), and Diabetes Mellitus (DM). In the NGR group, the CTI index demonstrated a robust and independent association with cognitive impairment risk. In the fully adjusted Model 3, each unit increase in CTI was associated with a 24.6% increased risk of cognitive impairment (HR = 1.246, 95% CI: 1.124–1.381, P < 0.001). Compared to the lowest quartile (Q1), participants in the highest quartile (Q4) had a significantly higher risk of cognitive impairment (HR = 1.582, 95% CI: 1.246–2.008, P < 0.001).

**Table 5:** Association between CTI and the risk of incident cognitive impairment according to glucose metabolic states.

Categories	Event, n%	Model 1		Model 2		Model 3	
		HR (95%)	P value	HR (95%)	P value	HR (95%)	P value
NGR							
Continuous variable per unit	823 (38.4)	1.198 (1.100-1.304)	<0.001	1.240 (1.123-1.370)	<0.001	1.246 (1.124-1.381)	<0.001
Quartile							
Q1	182 (29.8)	Ref.		Ref.		Ref.	
Q2	220 (38.8)	1.246 (1.023-1.517)	0.029	1.337 (1.064-1.681)	0.013	1.287 (1.020-1.623)	0.033
Q3	215 (40.3)	1.296 (1.063-1.579)	0.010	1.349 (1.068-1.702)	0.012	1.305 (1.031-1.653)	0.027
Q4	206 (47.7)	1.505 (1.232-1.839)	<0.001	1.600 (1.266-2.022)	<0.001	1.582 (1.246-2.008)	<0.001
Pre-DM							
Continuous variable per unit	784 (38.5)	1.179 (1.085-1.281)	<0.001	1.217 (1.110-1.335)	<0.001	1.240 (1.124-1.368)	<0.001
Quartile							
Q1	140 (29.7)	Ref.		Ref.		Ref.	
Q2	182 (36)	1.121 (0.900-1.397)	0.309	1.079 (0.847-1.376)	0.538	1.074 (0.841-1.371)	0.568
Q3	205 (39.9)	1.257 (1.015-1.558)	0.036	1.234 (0.972-1.567)	0.085	1.234 (0.970-1.569)	0.087
Q4	257 (47.4)	1.512 (1.231-1.857)	<0.001	1.624 (1.294-2.037)	<0.001	1.687 (1.337-2.128)	<0.001
DM							
Continuous variable per unit	165 (41.6)	1.117 (0.957-1.304)	0.161	1.139 (0.950-1.366)	0.160	1.165 (0.932-1.457)	0.180
Quartile							
Q1	23 (37.7)	Ref.		Ref.		Ref.	
Q2	24 (34.3)	0.752 (0.424-1.332)	0.328	0.649 (0.341-1.236)	0.188	0.613 (0.318-1.181)	0.144
Q3	37 (38.5)	0.885 (0.526-1.489)	0.645	0.781 (0.444-1.374)	0.392	0.733 (0.406-1.320)	0.300
Q4	81 (47.6)	1.117 (0.703-1.776)	0.639	1.051 (0.629-1.755)	0.85	1.007 (0.571-1.775)	0.981

CTI quartiles:Q1 (<8.211), Q2 (8.211-8.759), Q3 (8.759-9.475), Q4 (>9.475). Model 1, No variables adjusted; Model 2, adjusted for age, BMI, SBP, DBP, gender, educational attainment, smoking habits, and drinking habits; Model 3, Added adjustment for hypertension, hyperlipidemia, TC, FPG, HbA1c and heart disease on top of Model 2. HR, Hazard ratio. NGR, normoglycemic; Pre-DM, prediabetic; DM, diabetes mellitus.

A similar strong association was observed in the Pre-DM group. In Model 3, the CTI index remained a significant predictor of cognitive impairment (HR = 1.240, 95% CI: 1.124–1.368, P < 0.001). Notably, individuals in the highest CTI quartile (Q4) exhibited a 68.7% increased risk of cognitive impairment compared to those in Q1 (HR = 1.687, 95% CI: 1.337–2.128, P < 0.001).

**Figure 3:**
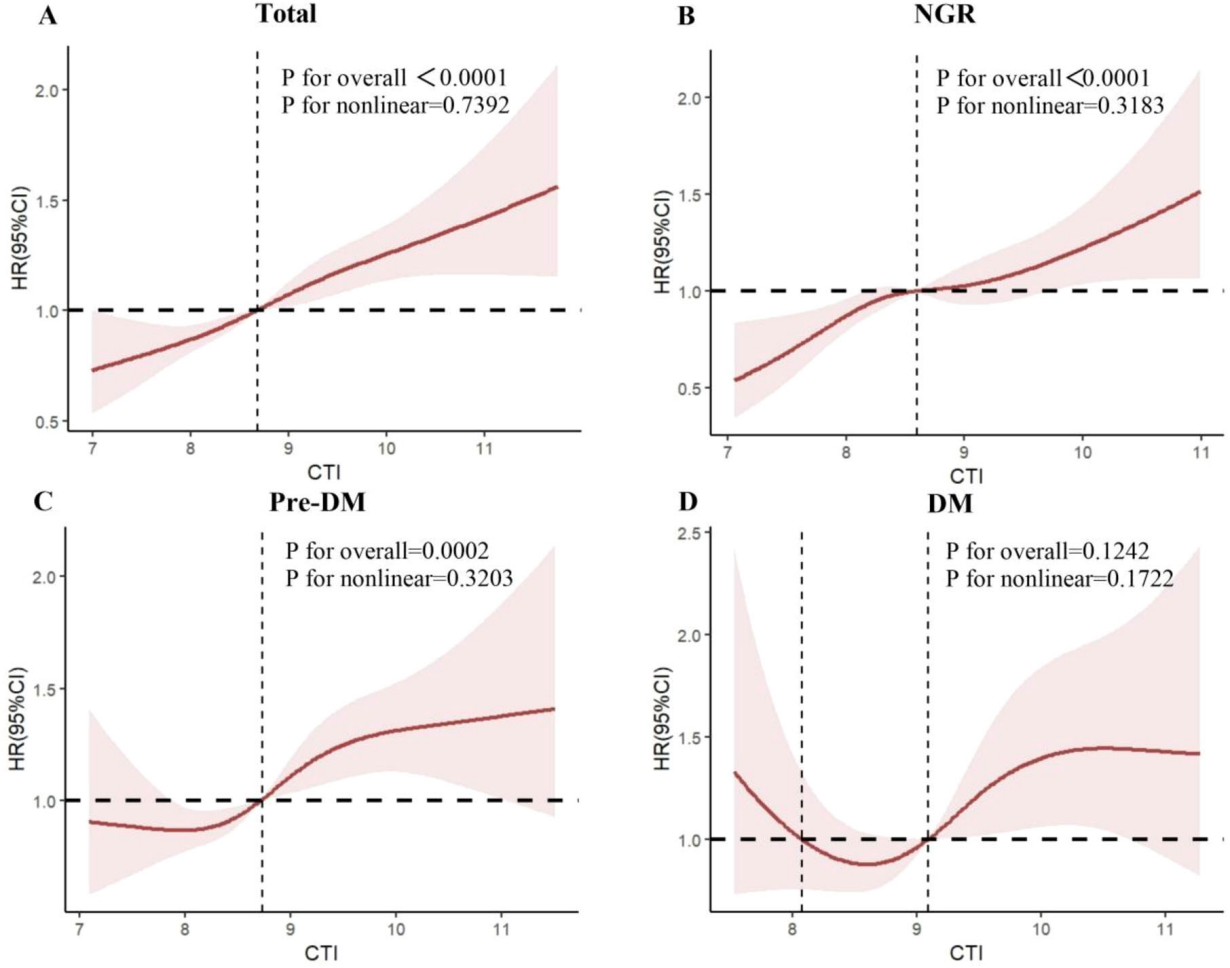
Restricted cubic spline (RCS) analysis of the nonlinear relationship between the C-reactive protein triglyceride glucose index (CTI) and the risk of cognitive impairment. Legend: restricted cubic spline curves wit 95% confidence intervals (shaded areas) depict the dose-response relationship between CTI and cognitive impairment risk. **(A)** In the overall study population, a linear positive association was observed, with MCI risk progressively increasing as CTI levels rose. **(B)** Among individuals with normal glucose regulation(NGR), a threshold effect was evident, with MCI risk remaining relatively stable below CTI≈8.7 increasing sharply beyond this point. **(C)** In the prediabetic (Pre-DM) group, a similar threshold pattern was observed, indicating heightened vulnerability at higher CTI levels. **(D)** In contrast, the diabetic (DM) subgroup demonstrated a U-shaped association, with the lowest MCI risk at intermediate CT levels, and higher risks at both low and high CTI extremes. The wide Cis at the upper range in the diabetic group indicates greater statistical uncertainty, likely due to limited sample size and population heterogeneity.

Interestingly, the association between CTI and cognitive impairment risk appeared to be attenuated in the DM group. Although the HRs for the continuous variable remained above 1.0 (e.g., Model 3: HR = 1.165, 95% CI: 0.932–1.457), they did not reach statistical significance (P = 0.180). Similarly, no significant risk increment was observed across the CTI quartiles in the DM subgroup. Formal interaction testing revealed no significant interaction between CTI and glycemic status (P for interaction = 0.534) (**Supplementary Table 6**). This indicates that the effect of CTI on cognitive impairment risk is statistically homogeneous across NGR, Pre-DM, and DM subgroups. Therefore, the non-significant association observed in the DM subgroup should be interpreted as reflecting reduced statistical power due to a smaller stratified sample size and wider confidence intervals, as well as a potential non-linear (U-shaped) relationship (**Figure 3**), rather than a true absence of association or effect modification by diabetes status.

#### 3.4.3 Dose-response relationships via RCS analysis stratified by glucose status

Restricted cubic spline (RCS) analysis revealed a threshold effect at CTI = 8.7. For participants with normal glucose regulation (NGR) or pre-diabetes, cognitive impairment risk increased sharply beyond this threshold, while T2DM participants exhibited a potential U-shaped association, with lowest risk at intermediate CTI levels (**Figure 3A**). This suggests that both excessively low or high metabolic–inflammatory burden may adversely affect cognitive health in diabetes.

#### 3.4.4 Sensitivity analysis excluding participants with functional disability

To address concerns that the operational definition of cognitive impairment might inadvertently include individuals with functional disability indicative of dementia, we conducted a sensitivity analysis excluding participants with an ADL score > 6 (n = 83 excluded; n = 4492 retained). In this functionally independent sub-cohort, the association between CTI and incident cognitive impairment remained robust and materially unchanged. In the fully adjusted model (Model 3), each unit increase in CTI was associated with an HR = 1.228 (95% CI: 1.153-1.308; P <0.001) increased risk of incident cognitive impairment. (**Supplementary Table 6**). These findings indicate that the observed association is not driven by the inclusion of individuals with advanced cognitive disability and support the validity of our operational definition for capturing early cognitive decline.

### 3.5 Development of a CTI-based prediction model

#### 3.5.1 Variable selection and model construction

To construct a robust and clinically applicable predictive model, a dual-stage variable selection strategy was implemented. First, LASSO regression was utilized to perform initial feature reduction from the pool of all candidate variables (including CTI, HbA1c, Age, etc.). Based on the optimal lambda identified through cross-validation, 10 variables were retained.

**Figure 4:**
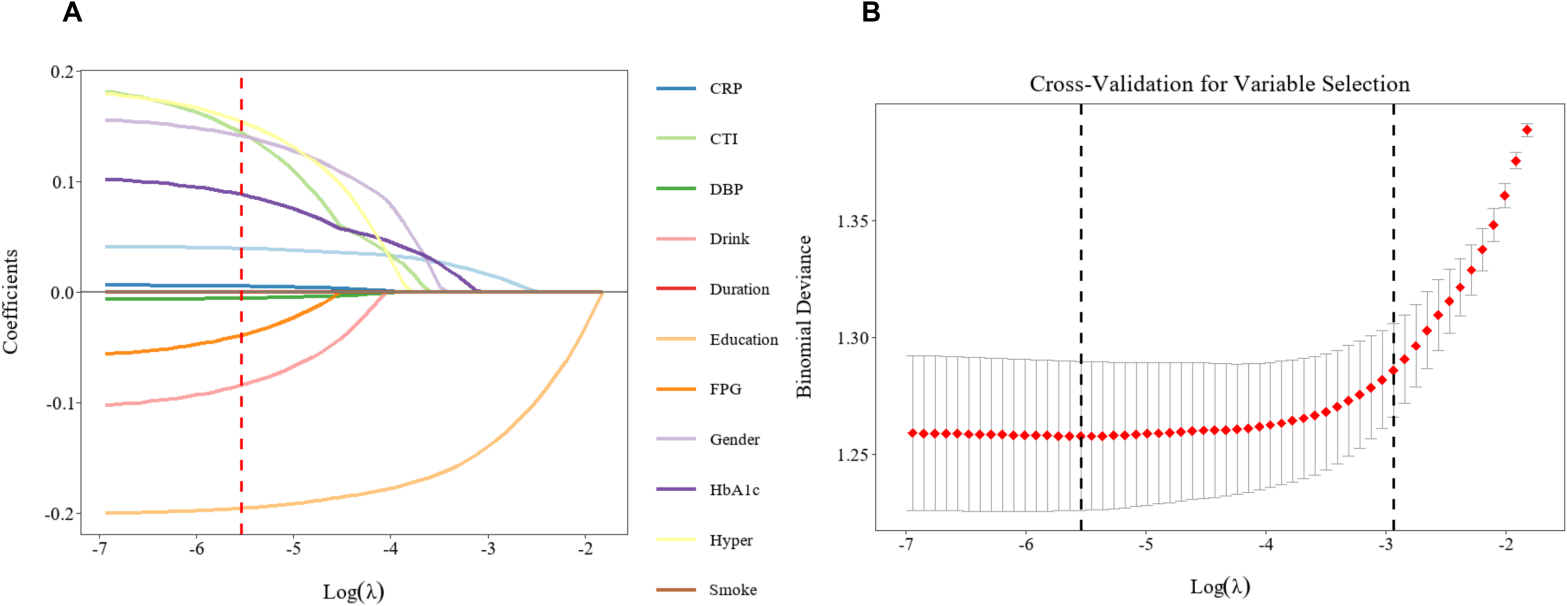
LASSO regression for variable selection. **(A)** LASSO coefficient paths of candidate predictors. Each colored line represents the trajectory of a regression coefficient as the regularization parameter λ varies. With increasing log(λ), coefficients are progressively shrunk toward zero. The vertical dashed line indicates the optimal λ selected by 10-fold cross-validation using the 1-SE criterion, at which 10 predictors with non-zero coefficients were retained. **(B)** Cross-validation error curve for LASSO regression. Binomial deviance is plotted as a function of log(λ). The red dots represent the average cross-validation error, and the vertical bars indicate the corresponding standard errors. The left vertical dashed line marks the λ value minimizing the deviance (λ_min), and the right vertical dashed line marks the largest λ within one standard error of the minimum (λ_1SE). The optimal λ for model parsimony (λ_1SE) was selected, as shown by the right dashed line. LASSO, least absolute shrinkage and selection operator; SE, standard error; HbA1c, glycated hemoglobin; FPG, fasting plasma glucose; DBP, diastolic blood pressure; CRP, C-reactive protein; CTI, C-reactive protein–triglyceride–glucose index.

A stepwise backward elimination procedure based on the Akaike Information Criterion (AIC) was then used to include these LASSO-selected variables into a multivariable logistic regression model. This refined step further optimized the model by excluding non-contributory factors, resulting in a final set of independent predictors: the CTI index, age, education, HbA1c and gender. This integrated approach ensured that the final model was both parsimonious and highly predictive, effectively balancing model fit with biological relevance. **Figure 4**.

#### 3.5.2 Model performance and clinical utility

**Figure 5:**
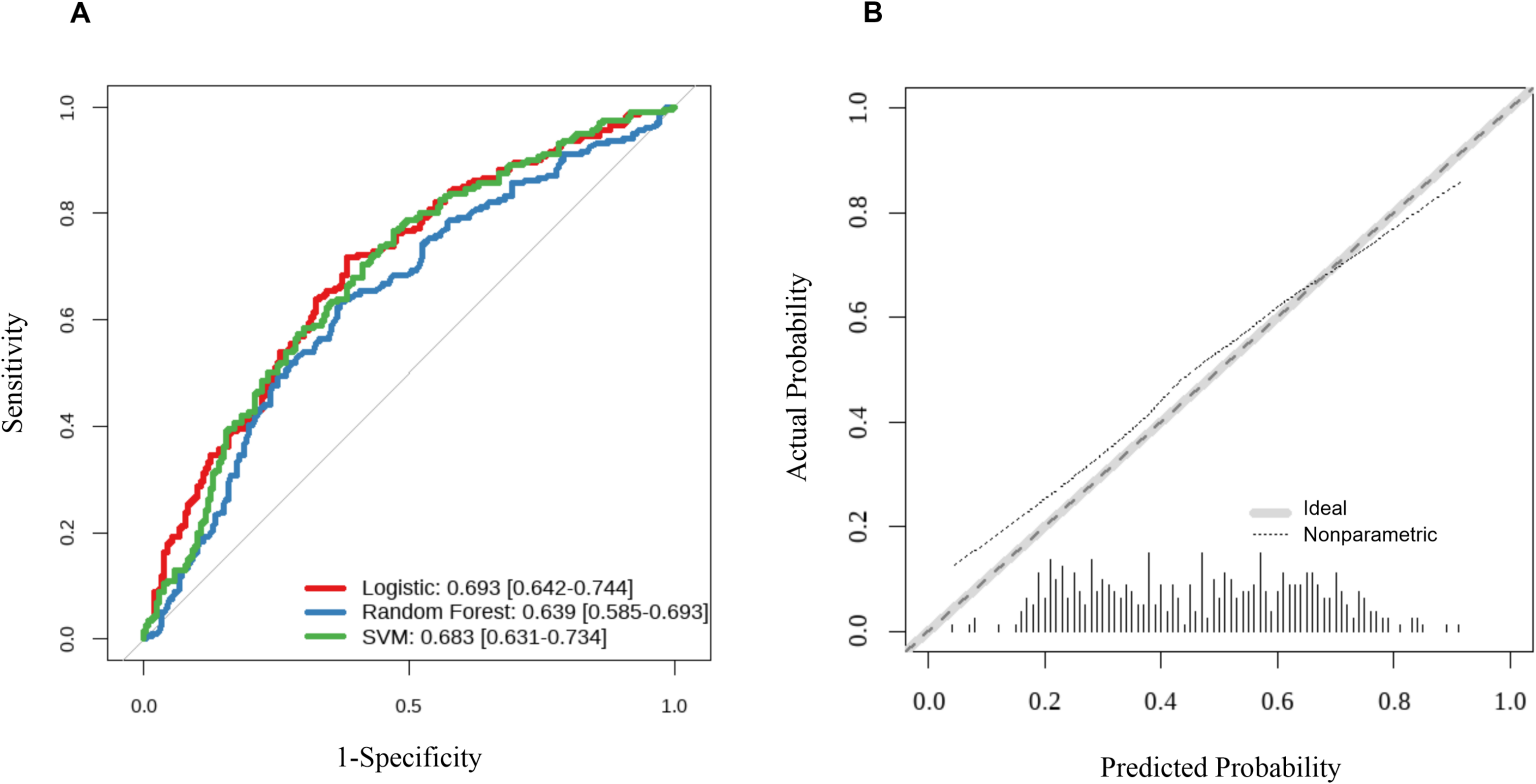
Performance evaluation of the prediction models in the validation set. **(A)** Receiver operating characteristic curves for three models. ROC curves compare the discriminatory ability of the logistic regression (red line), random forest (blue line), and support vector machine (green line) models in the internal validation set (n = 474). The area under the curve (AUC) with 95% confidence intervals for each model is shown in the legend. The logistic model achieved an AUC of 0.693 (95% CI: 0.642–0.744), the random forest model an AUC of 0.639 (95% CI: 0.585–0.693), and the SVM model an AUC of 0.683 (95% CI: 0.631–0.734). No statistically significant differences were detected among the three models (DeLong test, all P > 0.05). **(B)** Calibration plot of the logistic regression model. The calibration curve (dashed line) depicts the agreement between predicted probabilities and observed outcomes. Perfect calibration is represented by the grey dotted line (45° diagonal). Points close to the diagonal indicate good calibration. The Hosmer–Lemeshow goodness-of-fit test yielded a non-significant result (χ² = 8.24, P = 0.324), further supporting adequate calibration. ROC, receiver operating characteristic; AUC, area under the curve; CI, confidence interval; SVM, support vector machine.

As illustrated in the ROC curves (**Figure 5**), the Logistic Regression model achieved the highest discriminative ability with an AUC of 0.693 (95% CI: 0.642–0.744), slightly outperforming the SVM (AUC = 0.683) and significantly surpassing the Random Forest model (AUC = 0.639) (**Supplementary Table 3**). No statistically significant differences were detected among the three models. The calibration of the CTI-based Logistic Regression model was evaluated using a calibration plot (**Figure 5**). The non-parametric line (dotted line) remained in close proximity to the ideal 45-degree diagonal line (grey dashed line), indicating that the predicted probabilities of MCI were well-aligned with the actual observed events. This high level of calibration suggests that the model is reliable and does not suffer from significant overestimation or underestimation of risk.

**Figure 6:**
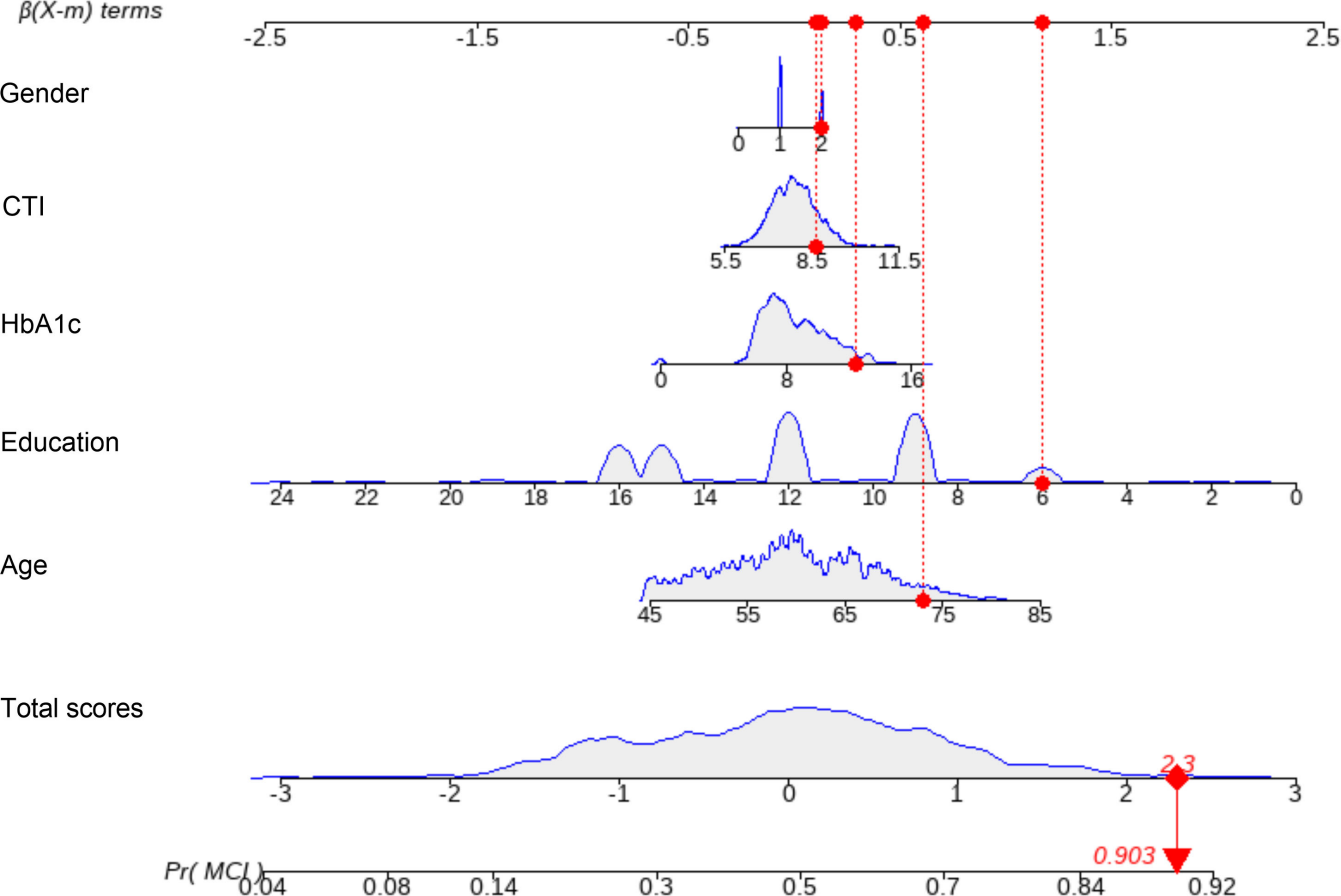
Nomogram for predicting mild cognitive impairment risk. The nomogram was developed based on the final multivariable logistic regression model in the training set (n = 1, 106). To estimate an individual’s probability of MCI, locate the value of each predictor on its corresponding axis, draw a vertical line upward to the “Points” scale to obtain the point contribution for that predictor, sum the points from all predictors, and locate the total points on the “Total Points” axis. A vertical line drawn downward from the total points to the “Probability of MCI” axis yields the individualized predicted risk. CTI, C-reactive protein–triglyceride–glucose index; HbA1c, glycated hemoglobin; MCI, mild cognitive impairment.

A nomogram was constructed based on the final model (**Figure 6**). The model incorporates five key variables: Gender, CTI index, HbA1c, Education level, and Age. In this scoring system, each predictor is assigned a point value based on its regression coefficient. For instance, a higher CTI index and HbA1c level, along with advanced age and lower educational attainment, contribute significantly to the cumulative risk score.

#### 3.5.3 Model interpretability

**Figure 7:**
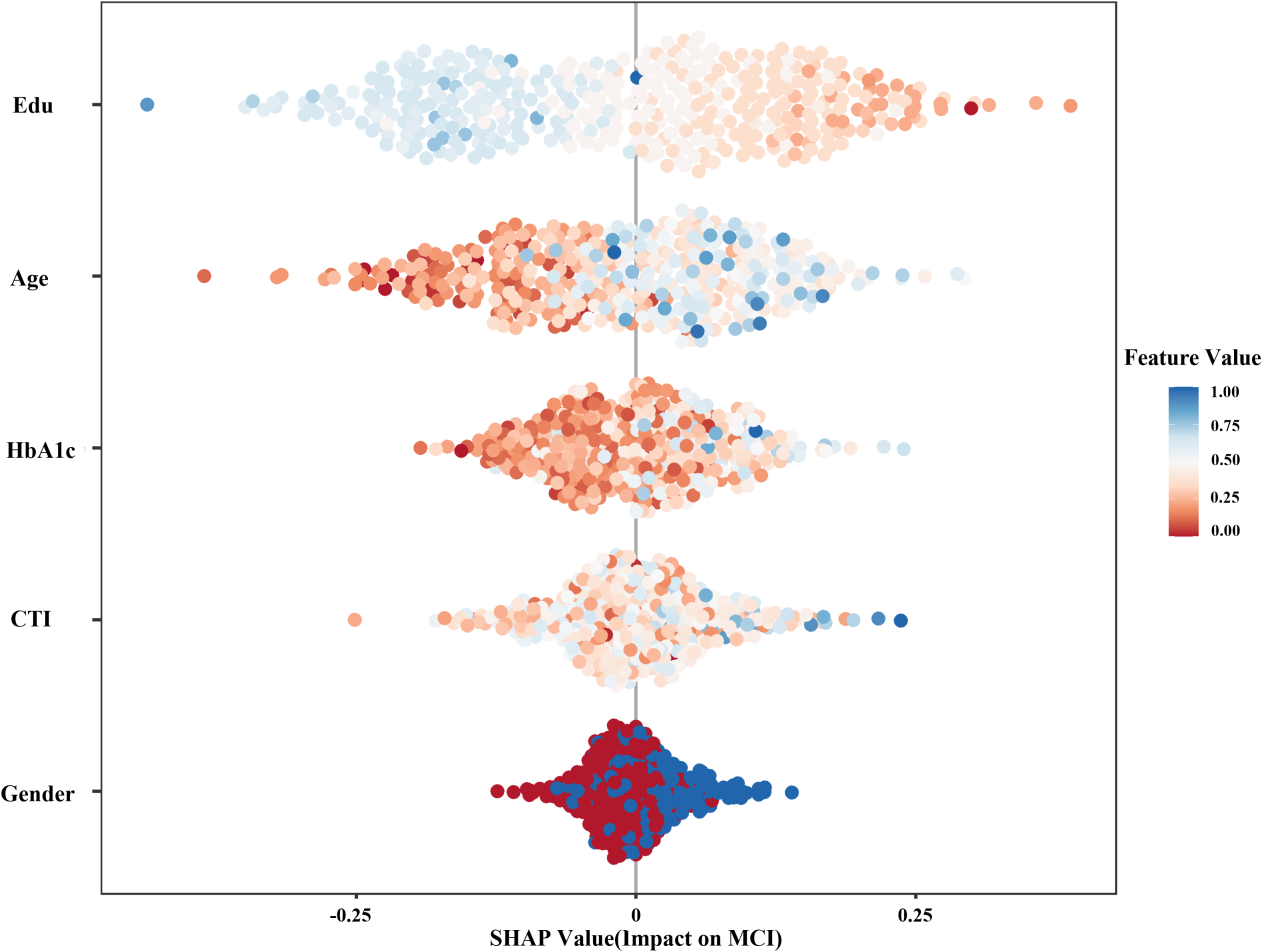
SHAP summary plot for feature importance in the prediction model. The SHAP (Shapley Additive Explanations) summary plot illustrates the relative contribution of each feature to the prediction of mild cognitive impairment (MCI). Features are ranked in descending order according to their mean absolute SHAP values, indicating overall importance. Each point represents an individual observation. The x-axis denotes the SHAP value, reflecting the impact of a given feature on the model output. Positive SHAP values indicate an increased risk of MCI, whereas negative values indicate a decreased risk. Color represents the feature value, with red indicating higher values and blue indicating lower values. The distribution of points along the x-axis reflects both the magnitude and direction of each feature’s effect.

The SHAP summary plot illustrates the relative importance and the direction of influence for the top predictors (**Figure 7**). Education and Age emerged as the most influential factors, followed closely by HbA1c and the CTI index.

Specifically, for the CTI index, the distribution of points shows that higher CTI values (represented by blue dots) are predominantly clustered on the right side of the zero line (positive SHAP values). This indicates that an increase in the CTI index consistently leads to a higher predicted risk of MCI. Similarly, higher levels of HbA1c and advanced Age were positively associated with MCI risk. In contrast, higher educational attainment (blue dots on the left) demonstrated a strong protective effect, significantly lowering the SHAP value and the corresponding likelihood of MCI.

## 4 Discussion

In this study, we found that the C-reactive Protein-Triglyceride Glucose index (CTI) was significantly associated with mild cognitive impairment (MCI).This association was consistent overall in both cross-sectional analyses and longitudinal validation in the CHARLS cohort, supporting its robustness and generalizability of our findings.

However, in subgroup analyses of the CHARLS cohort, the association between CTI and cognitive impairment did not reach statistical significance within the diabetes subgroup. This observation should be interpreted with caution and does not necessarily indicate a true biological inconsistency. Taken together, these subgroup nuances emphasize the importance of methodological considerations while supporting the overall association of CTI with cognitive impairment across populations.

Several factors may explain this finding. First and foremost, the interaction analysis revealed no significant modification by glycemic status (P for interaction = 0.534), indicating that the overall association is statistically consistent across NGR, Pre-DM, and DM subgroups. The non-significant finding in the DM subgroup is likely attributable to reduced statistical power due to a smaller stratified sample size and wider confidence intervals, rather than qualitative difference in the underlying biology. Second, there are notable differences in population characteristics between the two cohorts. For example, in the CHARLS cohort, the diabetes subgroup may exhibit heterogeneity in terms of disease duration, glycemic control and treatment methods compared with diabetic patients in the hospital cohort. Such heterogeneity may introduce additional noise and reduce the precision of the effect estimates.

Furthermore, the restricted cubic spline analysis revealed a potential U-shaped association in the DM subgroup (**Figure 3**), indicating that the relationship between CTI and cognitive impairment may not be adequately captured by a linear model. This non-linear pattern may reflect the competing risks of malnutrition, frailty, and hypoglycemia at low CTI levels—all recognized independent risk factors for cognitive decline in older diabetic populations (23)—alongside the detrimental effects of systemic inflammation at high CTI levels. A standard linear Cox regression model is inherently less sensitive to detecting associations when the true relationship is non-monotonic. This U-shaped pattern suggests that both excessively low and high metabolic-inflammatory burdens may adversely affect cognitive health in diabetes, warranting further investigation.

The attenuated linear association in the diabetes subgroup warrants a deeper exploration across biological, clinical, and methodological dimensions.

Biologically, individuals with established diabetes in both cohorts are expected to share similar underlying metabolic-inflammatory and neurodegenerative processes (4, 5). There is no strong evidence to suggest that the fundamental biological mechanisms linking metabolic-inflammatory burden and cognitive decline differ between the hospital-based and community-based diabetes populations (24). Therefore, the observed differences in results are unlikely to be attributable to inherent biological disparities, and are more plausibly explained by differences in study design, statistical modeling, or sample characteristics.

Clinically, several diabetes-specific factors may confound or modify the CTI–cognition association. First, medication regimens vary widely—some agents e.g., SGLT2 inhibitors (25, 26), GLP-1 receptor agonists (27) may exert neuroprotective effects independent of their glucose-lowering properties, while others (e.g., insulin, sulfonylureas) increase a risk of hypoglycemia, which is an established independent risk factor for dementia (23). These treatment-related effects could introduce substantial heterogeneity, diluting the observed linear association. Second, diabetes duration and glycemic variability—not fully captured in a community-based cohort like CHARLS—may be more relevant to cognitive outcomes than a single time-point metabolic-inflammatory index. Third, the presence of diabetic complications (nephropathy, neuropathy, cardiovascular disease) adds competing pathophysiological pathways that complicate the CTI–cognition relationship.

Methodologically, the null linear finding in the CHARLS-DM subgroup must be interpreted with caution. The stratified sample size was relatively modest, limiting statistical power. As previously shown, the non-linear association in the diabetes subgroup indicates that a linear Cox model may not fully capture the underlying relationship. Additionally, competing risk of mortality in older diabetic participants cannot be ignored; individuals with the highest metabolic-inflammatory burden may die before developing measurable cognitive impairment, leading to an underestimation of the true effect (survival bias). Finally, the operational definition of cognitive impairment in CHARLS may capture a relatively heterogeneous outcome in the DM subgroup, which could contribute to variability in effect estimates.

Taken together, the null finding in the DM subgroup does not imply that CTI is irrelevant in diabetes. Rather, its prognostic value may depend on clinical context, and more nuanced modeling approaches—including non-linear and competing risk frameworks—may be required. Further studies with larger diabetes-specific samples, repeated CTI measurements, and detailed treatment information are warranted to clarify these complex relationships.

The present findings should be situated within the broader context of evolving research on metabolic-inflammatory biomarkers for cognitive impairment. Traditional single markers—including HbA1c, fasting plasma glucose, and individual lipid parameters—have shown inconsistent associations with cognitive outcomes in observational studies (28–30). This inconsistency likely reflects the multifactorial nature of diabetes-related cognitive decline, which arises from the interplay of insulin resistance, dyslipidemia, and chronic low-grade inflammation, rather than from any single metabolic perturbation (4, 5).To better capture this multidimensional pathophysiology, several composite indices have been proposed. The triglyceride–glucose (TyG) index, a simple product of fasting glucose and triglycerides, has emerged as a reliable surrogate of insulin resistance and has been linked to an increased risk of cognitive impairment in both diabetic and general populations (7). The systemic immune-inflammation index (SII) and related markers have also been investigated, with evidence suggesting that integrating inflammatory parameters with metabolic measures may improve risk discrimination (31, 32). However, most existing composite indices focus predominantly on either metabolic dysregulation or inflammation, without fully integrating both pathways into a single measure. The C-reactive protein–triglyceride–glucose index (CTI) was specifically developed to address this gap by simultaneously reflecting systemic inflammation (hs-CRP) and insulin resistance (TyG) in a unified formula (11). As previously reported, CTI reflects systemic metabolic-inflammatory dysregulation, which may impact cognitive function (12–14).However, studies directly examining the association between CTI and cognitive impairment remain scarce, particularly in diabetic populations and in longitudinal settings with rigorous cognitive assessment. To our knowledge, this is the first study to: (i) evaluate CTI’s association with comprehensive neuropsychological outcomes in a clinically diagnosed T2DM cohort; (ii) validate the association prospectively in a nationally representative community-based cohort; and (iii) demonstrate incremental predictive value beyond the TyG index through formal reclassification analysis. Our findings therefore extend the existing CTI literature, which has largely focused on cardiovascular and hepatic outcomes, into the cognitive domain.

The mechanistic rationale for CTI’s predictive utility in cognitive outcomes is biologically plausible. Insulin resistance impairs cerebral glucose metabolism and promotes tau hyperphosphorylation and amyloid-β accumulation (4), while systemic inflammation compromises blood-brain barrier integrity and activates microglial-mediated neuroinflammation (33). Furthermore, hyperglycemia exacerbates advanced glycation end-products (AGEs) accumulation, oxidative stress, and mitochondrial dysfunction, all of which contribute to neuronal damage (34). These metabolic and inflammatory processes are closely interconnected, forming a “metabolic–inflammatory loop.” By integrating glycemic and inflammatory parameters, CTI may better capture this cumulative burden, providing a more comprehensive assessment of the underlying pathophysiology than either pathway marker alone. This integrated perspective may also explain the superior reclassification performance of CTI over the TyG index observed in the present study (NRI = 0.132, P = 0.031).

Although CTI showed similar discriminative performance to the TyG index (AUC comparison, P = 0.135), it provided a modest but statistically significant improvement in risk reclassification, as reflected by both the continuous NRI (0.132, P = 0.031) and IDI (0.004, P = 0.035).

In the context of predicting mild cognitive impairment in type 2 diabetes, however, even modest improvements in reclassification may carry clinical relevance. Mild cognitive impairment is notoriously underdiagnosed in routine diabetes care (17), and any readily accessible tool that can refine risk stratification—shifting a subset of patients into a more appropriate risk category—could trigger earlier and more targeted cognitive evaluation. Because CTI is derived entirely from routinely measured, low-cost laboratory parameters (CRP, triglycerides, fasting glucose) (22), this incremental improvement comes at virtually no additional cost or testing burden, making its cost-effectiveness profile exceptionally favorable for population-level screening (35).

From a mechanistic standpoint, the incremental reclassification provided by CTI over TyG likely reflects the additional dimension of systemic inflammation that CTI captures. While the TyG index serves as a reliable surrogate for insulin resistance, it does not account for the chronic low-grade inflammatory state that is independently implicated in the pathogenesis of diabetes-related cognitive decline (5). The NRI and IDI findings therefore provide formal statistical evidence that integrating inflammatory and metabolic pathways into a single index yields more nuanced risk information than considering either pathway in isolation, even though the magnitude of this improvement, when added to a well-calibrated base model, is necessarily limited.

Nevertheless, we caution against overinterpreting this incremental improvement. The modest NRI indicates that CTI should be viewed as a complementary marker rather than a standalone breakthrough predictor. Its clinical utility is likely maximized when embedded within a multivariable prediction model (as demonstrated by our nomogram) rather than used in isolation. Future studies with larger sample sizes and independent external validation cohorts are needed to determine whether the observed reclassification improvement can be replicated and translated into tangible clinical benefits, such as earlier intervention or reduced progression to dementia.

Further, CTI was more strongly associated with delayed memory than global cognition, consistent with the hippocampus’ particular vulnerability to metabolic and inflammatory insults (36). The association between CTI and MCI was also more pronounced in individuals aged ≥60 years, aligning with the concept of “inflammaging, “ where combined effects of metabolic dysfunction and age-related immune changes accelerate neuronal damage and reduce cognitive reserve (37, 38). These findings suggest that preventive strategies should extend beyond glucose control to include lifestyle interventions such as physical activity and adherence to anti-inflammatory dietary patterns (39).

Several limitations should be acknowledged. First, although the findings were validated in an external cohort, causal inference is limited due to the observational design.

Second, CTI is derived from routine clinical markers and may not fully capture the complexity of neuroinflammation. In addition, the relatively modest improvement observed in reclassification metrics suggests that further refinement of predictive models is needed before clinical implementation. Future studies incorporating more specific biomarkers, such as glial fibrillary acidic protein (GFAP), may provide additional insights into underlying mechanisms (24). Third, residual confounding cannot be entirely excluded. Fourth, the definition of cognitive impairment in the CHARLS cohort was based on performance-based criteria rather than formal clinical diagnosis, which may have led to some degree of outcome misclassification. Nevertheless, our sensitivity analyses excluding participants with functional limitations yielded consistent findings, and interaction analyses confirmed effect homogeneity across glycemic subgroups. These results support the robustness of the observed association and justify the use of this operational definition in the context of a large-scale epidemiological study.

In summary, CTI, as an integrated marker of metabolic and inflammatory burden, is consistently associated with MCI risk in both diabetic and general populations. By bridging metabolic and immune pathways, CTI provides modest incremental information beyond traditional markers may serve as a complementary approach for identifying individuals at increased risk, although its clinical utility requires further validation and intervention in high-risk individuals.

## Data Availability

The datasets presented in this study can be found in online repositories. The names of the repository/repositories and accession number(s) can be found below: http://charls.pku.edu.cn/.
