# Supplementary material for: A multi-stage validation of the C-reactive protein–triglyceride–glucose index for predicting mild cognitive impairment: evidence from clinical and nationwide prospective cohorts
Source: Front Endocrinol (Lausanne). 2026 May 19;17:1841431. doi: 10.3389/fendo.2026.1841431 (PMC13225990; doi:10.3389/fendo.2026.1841431)
Supplement: Supplementary file 1 [file DataSheet1.docx]

**Supplementary Material**

Table S1 Multivariable Logistic Regression Analysis of Risk Factors for MCI

| **Variable** | **Model 1 (TyG)** | | | **Model 2 (CTI)** | | |
| --- | --- | --- | --- | --- | --- | --- |
|  | β | OR (95% CI) | P | β | OR (95% CI) | P |
| Age | 0.049 | 1.050 (1.034–1.066) | < 0.001 | 0.049 | 1.051 (1.035–1.067) | < 0.001 |
| Education | -0.196 | 0.822 (0.794–0.851) | < 0.001 | -0.195 | 0.823 (0.795–0.852) | < 0.001 |
| Duration | -0.001 | 0.999 (0.985–1.014) | 0.899 | 0 | 1.000 (0.986–1.015) | 0.998 |
| HbA1c | 0.05 | 1.051 (0.999–1.106) | 0.055 | 0.045 | 1.046 (0.994–1.101) | 0.081 |
| TyG | 0.147 | 1.159 (0.982–1.367) | 0.08 | — | — | — |
| CTI | — | — | — | 0.196 | 1.217 (1.056–1.402) | 0.007 |

**Note:** Model 1 included age, education, diabetes duration, HbA1c, and the TyG index. Model 2 included the same covariates but replaced the TyG index with the CTI.

*Abbreviations: TyG, triglyceride-glucose index; CTI, C-reactive protein–triglyceride–glucose index; OR, odds ratio; CI, confidence interval.*

Table S2 Baseline Characteristics Table in a Longitudinal Study

| **Characteristics** | **Total** | **Quartiles of CTI** | | | | **P value** |
| --- | --- | --- | --- | --- | --- | --- |
|  |  | **Q1** | **Q2** | **Q3** | **Q4** |  |
| **Number** | **4575** | **1143** | **1143** | **1144** | **1144** |  |
| Age,years | 58.86±9.05 | 57.05±8.49 | 58.49±8.90 | 59.46±8.82 | 59.72±8.96 | ＜0.001 |
| Female,n(%) | 2476(54.1) | 616(24.9) | 614(24.8) | 598(24.2) | 648(24.6) | 0.199 |
| BMI,kg/m^2^ | 24.07±3.54 | 22.75±3.75 | 24.31±3.11 | 24.68±2.12 | 24.52±4.37 | 0.258 |
| **Education level,n(%)** |  |  |  |  |  | 0.277 |
| ≤12 years | 3465(82) | 847(81.2) | 863(82.7) | 871(81.3) | 884(82.6) |  |
| ＞12 years | 760(18) | 196(18.8) | 180(17.3) | 200(18.7) | 184(17.2) |  |
| **Health,n(%)** |  |  |  |  |  | 0.005 |
| Poor | 157(3.4) | 30(2.6) | 34(3) | 41(3.6) | 52(4.6) |  |
| Fair | 913(20) | 205(17.9) | 210(18.4) | 225(19.7) | 273(23.9) |  |
| Good | 2379(52.1) | 612(53.5) | 624(54.6) | 593(51.9) | 550(48.2) |  |
| Very good and above | 1121(24.6) | 297(25.9) | 274(24) | 283(24.8) | 267(23.4) |  |
| **Smoking status,n(%)** |  |  |  |  |  | 0.141 |
| Never or former | 3366(77.2) | 832(76.8) | 811(75.4) | 848(77.2) | 875(79.5) |  |
| Current | 992(22.8) | 252(23.2) | 264(24.6) | 251(22.8) | 225(20.5) |  |
| **Drinking status,n(%)** |  |  |  |  |  | 0.073 |
| Never or former | 2886(63.1) | 694(60.7) | 712(62.3) | 752(65.8) | 728(63.7) |  |
| Current | 1687(36.9) | 252(39.3) | 264(37.7) | 251(34.2) | 225(36.3) |  |
| Hypertension ,n(%) | 1228(27) | 219(17.8) | 264(21.5) | 339(27.6） | 406(33.1) | ＜0.001 |
| Diabetes ,n(%) | 397(6.9) | 61(5.3) | 70(6.1) | 96(8.4) | 170(14.9) | ＜0.001 |
| Dyslipidemia,n(%) | 518(11.5) | 86(7.6) | 118（10.5) | 158(14.1) | 156(13.9) | ＜0.001 |
| Heart disease,n(%) | 568(12.5) | 106(9.3) | 132(11.6) | 154(13.6) | 176(15.5) | ＜0.001 |
| FPG,mg/dl | 102.42(94.68,112.68) | 100.26(92.88,109.08) | 101.88(94.86,111.42) | 103.14(95.04,114.12) | 104.4（95.94,117.18） | ＜0.001 |
| Hba1c,% | 5.28±0.82 | 5.08±0.47 | 5.14±0.51 | 5.23±0.6 | 5.67±1.26 | ＜0.001 |
| TC,mg/dl | 194±38.32 | 189.71±37.18 | 193.47±37.11 | 198.54±39.42 | 196.28±39 | ＜0.001 |
| HDL-C,mg/dl | 50.65(39.82,59.15) | 54.46(43.68,63.02) | 51.38(40.98,59.53) | 49.04(38.66,56.44) | 47.74(36.72,55.67) | ＜0.001 |
| LDL-C,mg/dl | 117.95(94.33,139.18) | 113.17(91.23,134.15) | 117.74(95.10,137.53) | 121.45(97.42,144.11) | 119.45(94.33,141.88) | ＜0.001 |
| CRP,mg/l | 2.66(0.56,2.10) | 0.63(0.34,0.75） | 0.85(0.56,1.37) | 2.07(0.81,2.30） | 6.79(1.34,5.63) | ＜0.001 |
| Cognitive scores | 13.19±2.64 | 13.27±2.57 | 13.21±2.69 | 13.12±2.66 | 13.14±2.65 | 0.544 |
| Immediate memory | 4.41±1.56 | 4.46±1.51 | 4.45±1.59 | 4.41±1.57 | 4.33±1.56 | 0.205 |
| Delayed memory | 3.53±1.8 | 3.65±1.77 | 3.57±1.82 | 3.51±1.83 | 3.41±1.81 | 0.013 |
| GMS,n(%) |  |  |  |  |  | ＜0.001 |
| NGR | 2142(46.8) | 610(53.3) | 567(49.6) | 534(46.7) | 431(37.7) |  |
| Pre -DM | 2034(44.5) | 473(41.3) | 506(41.3) | 513(44.9) | 542（47.4） |  |
| DM | 397(8.7) | 61(5.3) | 70(6.1) | 96(8.4) | 170（14.9） |  |

Table S3 Comparison of Predictive Performance Among Different Models in the Validation Set

| **Model Type** | **AUC** | **Accuracy** | **Recall** | **Precision** | **F1-Score** | **Brier Score** |
| --- | --- | --- | --- | --- | --- | --- |
| Logistic Regression | 0.6926 | 0.6466 | 0.6231 | 0.6526 | 0.6375 | 0.2268 |
| Random Forest | 0.6388 | 0.599 | 0.5829 | 0.601 | 0.5918 | 0.2458 |
| Random Forest（tuned） | 0.6785 | 0.6391 | 0.6181 | 0.6406 | 0.6292 | 0.2291 |
| SVM | 0.6766 | 0.6341 | 0.598 | 0.6432 | 0.6198 | 0.2272 |
| SVM（tuned） | 0.6775 | 0.6355 | 0.6012 | 0.6445 | 0.6221 | 0.227 |

**Note:** Model performance was evaluated in the internal validation set (n = 474). Hyperparameter tuning for random forest and support vector machine models was performed using 10‑fold cross‑validation in the training set. AUC, area under the receiver operating characteristic curve; SVM, support vector machine. The logistic regression model demonstrated comparable performance to the tuned machine learning models, with no statistically significant differences in AUC detected by the DeLong test (all P > 0.05). The Brier score, ranging from 0 to 1, assesses the overall accuracy of probabilistic predictions, with lower values indicating better calibration.

Table S4 Multivariable Logistic Regression Analysis for MCI Prediction in the Training Set

| **Associated factors** | **β** | **OR(95%CI)** | **P value** |
| --- | --- | --- | --- |
| Age | 0.047 | 1.048 (1.030-1.067) | ＜0.001 |
| HbA1c | 0.109 | 1.116 (1.041-1.198) | 0.0023 |
| CTI | 0.239 | 1.270 (1.043-1.551) | 0.0178 |
| Educational attainment | -0.202 | 0.816 (0.781-0.853) | ＜0.001 |
| Gender | 0.204 | 1.220 (0.928-1.618) | 0.151 |

**Note:**The final model was derived from multivariable logistic regression using variables selected by LASSO regression followed by backward stepwise selection in the training set (n = 1,106). All variables listed were included in the final model regardless of statistical significance to maintain clinical interpretability. CTI remained a significant independent predictor after full adjustment (OR = 1.270, 95% CI: 1.043–1.551, P = 0.0178).

Table S5. Comparison of the CTI index as a continuous variable in primary and sensitivity analyses

| **Analysis Type** | **Participants (N)** | **Hazard Ratio (HR)** | **95% Confidence Interval (CI)** | **P value** |
| --- | --- | --- | --- | --- |
| Primary Analysis (Original) | 4,575 | 1.245 | 1.164 – 1.332 | < 0.001 |
| Sensitivity Analysis (Refined) | 4,492 | 1.228 | 1.153-1.308 | < 0.001 |

**Note:**

Primary Analysis: Includes the full longitudinal cohort as reported in the main results (Table 4).

Sensitivity Analysis: Excludes 83 participants who met the criteria for suspected dementia during follow-up.

Exclusion Criteria for Dementia:

(a) Decline in cognitive score > 1 SD;

(b) ADL score > 6;

(c) Self-reported or physician-diagnosed dementia.

Adjustment Factors: Both models are adjusted for Age, Gender, Education, BMI, Smoking, Drinking, Hypertension, SBP, DBP, TC, FPG, HbA1c, and Heart disease.

**Abbreviations:** CTI, C-reactive protein–triglyceride glucose index; MCI, mild cognitive impairment.

Table S6. Subgroup analyses of the association between CTI index quartiles and incident MCI risk.

|  | **Quartiles of CTI** | | | | **P for interaction** |
| --- | --- | --- | --- | --- | --- |
|  | **Q1** | **Q2** | **Q3** | **Q4** |  |
| **Gender** |  |  |  |  | 0.08 |
| Female | 1(Ref.) | 1.107(0.901-1.360) | 1.096(0.892-1.346) | 1.582(1.309-1.911) |  |
| Male | 1(Ref.) | 1.194(0.981-1.454) | 1.384(1.143-1.676) | 1.4(1.154-1.700) |  |
| **BMI** |  |  |  |  | 0.571 |
| ＜24kg/m^2^ | 1(Ref.) | 1.244(0.952-1.627) | 1.261(0.976-1.628) | 1.530(1.191-1.966) |  |
| ≥24kg/m^2^ | 1(Ref.) | 1.142(0.951-1.371) | 1.287(1.067-1.552) | 1.586(1.329-1.894) |  |
| **Hypertension** |  |  |  |  | 0.611 |
| YES | 1(Ref.) | 1.212(0.895-1.641) | 1.212(0.895-1.641) | 1.629(1.226-2.164) |  |
| NO | 1(Ref.) | 1.155(0.985-1.353) | 1.273(1.086-1.492) | 1.442(1.232-1.687) |  |
| **GMS** |  |  |  |  | **0.534** |
| NGR | 1(Ref.) | 1.246(1.023-1.517) | 1.296(1.063-1.579) | 1.505(1.232-1.839) |  |
| Pre-DM | 1(Ref.) | 1.121(0.900-1.397) | 1.257(1.015-1.558) | 1.512(1.231-1.857) |  |
| DM | 1(Ref.) | 0.752(0.424-1.332) | 0.885(0.526-1.489) | 1.117(0.703-1.776) |  |

**Note:** P for interaction between CTI (continuous) and glycemic status (NGR, Pre-DM, DM) = 0.534, indicating no statistically significant effect modification by glucose metabolic state.

**Abbreviations:** CTI, C-reactive protein–triglyceride glucose index; MCI, mild cognitive impairment; BMI, body mass index; SBP, systolic blood pressure; DBP, diastolic blood pressure; TC, total cholesterol; FPG, fasting plasma glucose; HbA1c, glycated hemoglobin; GMS, glycemic metabolic status.

Figure S1 Forest plot of subgroup analyses for the accociation between the C-reactive protein-triglyceride glucose index (CTI) and risk of mild cognitive impairment (MCI).

**Legend:** The forest plot illustrates hazards ratios (HR) and 95% confidence intervals (CIs) derives from Cox proportional hazards models assessing the predictive role of CTI for incident MCI across predefined subgroups. Subgroup stratifications included age (<60 vs.≥60 years), sex (male vs. female), body mass index (<24 vs.≥24kg/m^2^) ，hypertension status (yes vs. no), and diabetes duration (<5 vs.≥5years). Acorss most subgroups, elevated CTI was significantly associated with higher risk of MCI, with particularly strong associations observed among participants aged≥60 years and with diabetes duration ≥5 years. P-values for interaction were >0.05 in most subgroups, suggesting that the predictive role of CTI is broadly consistent across different demographic and clinical profile.


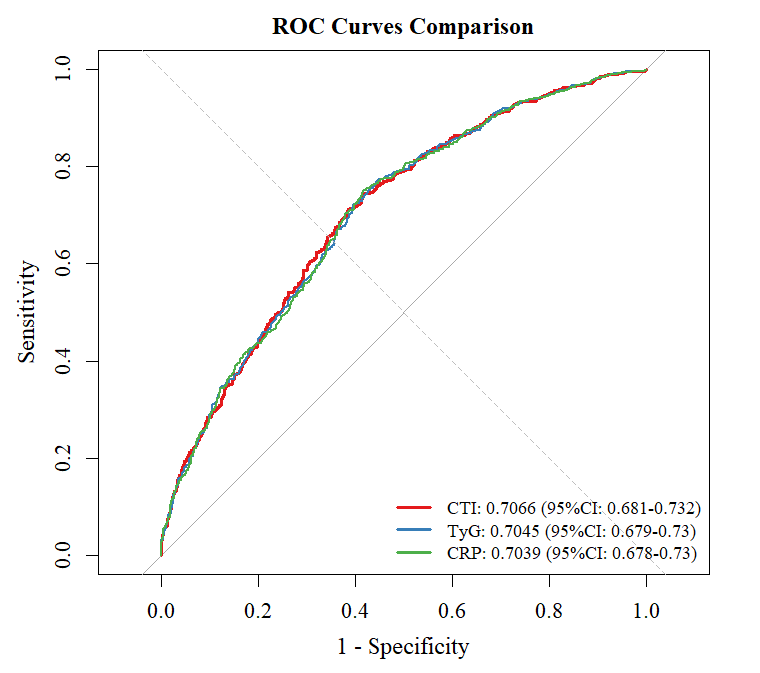


Figure S2 Receiver operating characteristic (ROC) curves of different markers for predicting MCI.

**Legend:**

The ROC curves illustrate the discriminative ability of the three models: the TyG-based model (blue line; AUC = 0.7045, 95% CI: 0.679–0.730), the CRP-based model (green line; AUC = 0.7039, 95% CI: 0.678–0.730), and the CTI-based model (red line; AUC = 0.7066, 95% CI: 0.681–0.732). All models were adjusted for age, education, diabetes duration, and HbA1c. The CTI-based model demonstrated the highest AUC among the three markers.

*Abbreviations: ROC, receiver operating characteristic; AUC, area under the curve; CI, confidence interval; TyG, triglyceride-glucose index; CRP, C-reactive protein; CTI, combined TyG-inflammation index; MCI, mild cognitive impairment; T2DM, type 2 diabetes mellitus.*

**
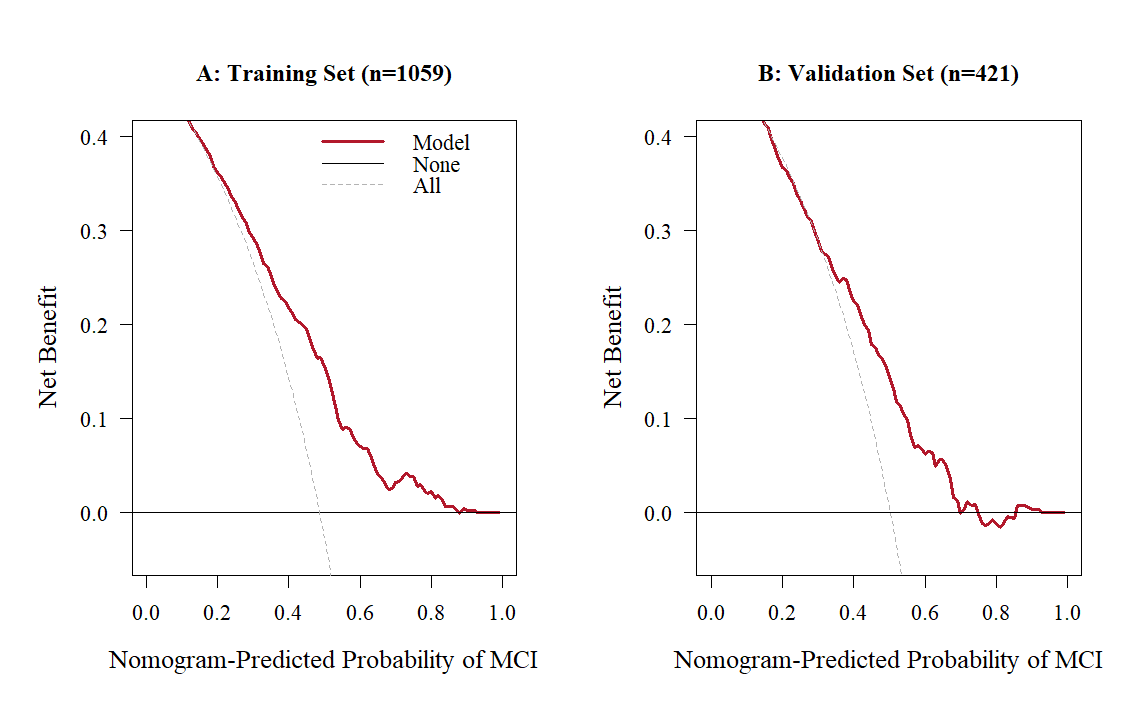
**

Figure S3 Decision Curve Analysis for the Logistic Regression Model in the Training and Validation Sets

**Legend：**Decision curves evaluate the clinical utility of the final logistic regression model by plotting net benefit against threshold probabilities. The analysis was performed in both the training set (n = 1,059,left) and the internal validation set (n = 421,right). Across a range of threshold probabilities from 10% to 60%, the model demonstrated positive net benefit in both cohorts, indicating that using the model for clinical decision‑making would yield better outcomes than either treating all patients or treating none within this risk range. Net benefit is calculated as the proportion of true positives minus the proportion of false positives weighted by the odds of the threshold probability.
